# Trend tests for the evaluation of exposure-response relationships in epidemiological exposure studies

**DOI:** 10.1186/1742-5573-6-1

**Published:** 2009-03-06

**Authors:** Ludwig A Hothorn, Michael Vaeth, Torsten Hothorn

**Affiliations:** 1 Institute of Biostatistics, Leibniz University Hannover, D-30419, Hannover, Germany; 2 Department of Biostatistics, University of Aarhus, Vennelyst Boulevard 6, DK-8000, Aarhus C, Denmark; 3 Institut fuer Statistik, Ludwig Maximilians Universitaet Muenchen, Ludwigstrasse 33, D-80539, Munich, Germany

## Abstract

One possibility for the statistical evaluation of trends in epidemiological exposure studies is the use of a trend test for data organized in a 2 × *k *contingency table. Commonly, the exposure data are naturally grouped or continuous exposure data are appropriately categorized. The trend test should be sensitive to any shape of the exposure-response relationship. Commonly, a global trend test only determines whether there is a trend or not. Once a trend is seen it is important to identify the likely shape of the exposure-response relationship. This paper introduces a best contrast approach and an alternative approach based on order-restricted information criteria for the model selection of a particular exposure-response relationship. For the simple change point alternative *H*_1 _: *π*_1 _= ...= *π*_*q *_<*π*_*q*+1 _= ... = *π*_*k *_an appropriate approach for the identification of a global trend as well as for the most likely shape of that exposure-response relationship is characterized by simulation and demonstrated for real data examples. Power and simultaneous confidence intervals can be estimated as well. If the conditions are fulfilled to transform the exposure-response data into a 2 × *k *table, a simple approach for identification of a global trend and its elementary shape is available for epidemiologists.

## Introduction

Statistical trend analysis is an important component of epidemiological exposure studies. Here, "trend" simply means the demonstration of any monotone relationship between the response rate and the continuous exposure. For example, the association between all major types of childhood cancer and exposure to magnetic fields from high voltage installations was analyzed by Lausen et al. [1] using the data shown in Table 1, where the original continuous exposure data (Olsen et al., [2]) were categorized.

**Table 1:** Child cancer and magnetic fields

Exposure/*μ *Tesla	*j*	n_cancer_	n_no cancer_	n_j_	*p*_*j*_	*RR*_*j*1_
0–0.05	1	1698	4759	6457	0.263	-
0.051–0.101	2	0	9	9	0	0.000
0.101-0.15	3	2	3	5	0.4	1.525
0.151-.20	4	1	3	4	0.25	0.953
0.201-0.25	5	1	3	4	0.25	0.953
0.251-0.30	6	0	4	4	0	0.000
0.301-0.35	7	0	2	2	0	0.000
0.351-0.85	8	1	0	2	0.5	1.906
0.851-1.6	9	2	0	2	1	3.812
>1.61	10	2	0	2	1	3.812

(p_j _... estimated proportion, RR_j1 _... relative risk to unexposed)

Although this example is seriously unbalanced, real epidemiological exposure studies with many unexposed or low-exposure cases but few high-exposure cases can be found. The appropriate evaluation of such epidemiological exposure studies is a statistical challenge. Many similar examples can be found in the literature, e.g. a case-control study for respiratory cancer possibly caused by long-term exposure to coke oven emissions [3].

In exposure studies, an unexposed group, E_1_, is commonly compared with several exposure groups, E_2_,..., E_k_. The outcome of the study is the number of cases suffering from the disease being investigated, such as a specific tumor, and the number of observations without the disease (controls), i.e. the risk of disease in each category of exposure. One important objective in exposure epidemiology is causation; the demonstration of a global exposure-response relationship represents one of the causation criteria, according to Hill [4]. A global trend test leads to identification of a trend, whereas model selection allows inference of the likelihood of a particular elementary model.

The sampling strategy of epidemiological exposure studies is either a cohort study, in which a 2 × *k *contingency table represents the data, or a case-control study, in which two multinomial distributions are compared. However, the likelihood ratio test of identical multinomials against the elementary odds ratios alternative, for a sufficient total number of observations, is equivalent to the comparison of the *k *independent binomial proportions against a simple ordered alternative (Agresti and Coull, [5]; Hothorn et al., [6]). Therefore, it is appropriate to evaluate both designs by means of an asymptotic trend test for a 2 × *k *contingency table.

Numerous methods, including model-based (e.g. Royston et al., [7]) and test-based approaches (e.g. Dosemeci and Benichou, [8]), are used to analyze exposure-response relationships. A basic problem is that the shape of the exposure-response is unknown a priori and is an outcome of the study. However, the choice of model or test greatly depends on the shape of the exposure-response. Therefore, a broad class of models or tests should be used, but that, in turn, leads to a model selection dilemma. Model selection is an intricate component of statistical problems. Model selection in this case is not the objective, but is only a tool for identifying the correct trend from several possible elementary alternatives. An alternative hypothesis can be decomposed into its underlying elementary alternatives, e.g. the simple order alternative *H*_1_: *π*_1 _≤ *π*_2 _≤ *π*_3 _can be decomposed into the three elementary hypotheses $H_{1}^{1}$ : *π*_1 _= *π*_2 _<*π*_3_, $H_{1}^{2}$ : *π*_1 _<*π*_2 _= *π*_3_, $H_{1}^{3}$ : *π*_1 _<*π*_2 _<*π*_3_.

The *p*-value, a commonly used outcome of a trend test, is frequently insufficient for epidemiological studies. Information concerning the shape of the exposure-response and/or a measure of the magnitude of the effect, such as relative risks or odds ratios, is desirable for a significant trend. Thus, the level of the false positive decision rate (*α*) should be controlled. In addition, an approach with a minimum false negative decision rate (*β*) (respective maximum power *π *= 1 - *β*) for the global test decision and a maximum correct decision rate for the selected model should be identified. The correct classification rate, the proportion of correctly identified elementary alternatives, is used as a major performance measure later on.

The exposure in case-control studies is frequently measured on a continuous scale. Categorization at pre-selected cut-off points of a small number of ordered categories is common; for example, four categories of trihalomethane exposure (Jones et al., [9]), or three categories of lifetime dose of hair dye (Benavente et al., [10]). Inappropriately chosen cut-off points dramatically reduce the power of the trend test (Greenland, [11]). Some exposures are naturally grouped, for example 2–3 cups of coffee per day, by the impreciseness of the definitions, such as "cup" and "coffee" (Ascherio et al., [12]). An example of ordinal definition of the exposure is given in a case-control study of Norwegian nickel refinery workers (Grimsrud et al., [13]). The exposure-related associations between smoking-adjusted lung cancer rates and cumulative exposure to different forms of nickel used the categories "low," "medium," and "high."

The best approach, in terms of both power and interpretation, occurs when a single cut-off point exists and is known a priori, resulting in a two-sample test "above" vs. "below" the cut-off point. This is because an odds ratio and its one-sided confidence interval can be estimated. The trend test approach discussed here is designed for naturally grouped exposure with a single change point. For continuous exposure models a continuous covariate can be used. However, the choice of an appropriate model – such as linear, logistic, or other – remains open and model selection influences the inference.

In this paper, a trend test for the comparison of *k *ordered binomial proportions using a change point alternative is presented. Either a single change point is directly of interest or the change point alternative is pivotal, i.e. many other elementary monotone alternatives can be generated from it. The concept of multiple contrasts is used because of the simplicity and the availability of the distribution under the alternative. After a significant trend test, information is provided that determines which contrast was the "best," and therefore, which exposure-response shape describes the data most accurately. Alternatively, an information criterion-based approach for the likelihood ratio test under monotone order-restriction according to Anraku [14] is examined.

Therefore, the primary objective of this paper is not just describing the exposure-response relationship but also identifying the most likely elementary exposure-response model with a control of the false model classification rate.

## Analysis

### Global tests on exposure-response relationships

The number of diseased and healthy persons for each exposure group, Ej, are organized in the following 2 × *k *table, where Index 1 denotes the group without exposure.

**Table 2:** Principle of 2 by *k *tables for epidemiological exposure studies

	E_1_	....	E_k_	Total
Disease	n_11_	...	n_k1_	n_.1_
No disease	n_10_	...	n_k0_	n_.0_
Sample size	n_1._	...	n_k._	n_.._

The estimator for the proportions per exposure group is *p*_*j *_= *n*_*j*1_/*n*_*j*. _*j *= 1,..., *k*, the total is *p *= *n*_.1_/*n*.., and the expected values for the proportions are denoted as *π*_*j*_. The hypotheses system for a monotone order is:

*H*_0_: *π*_1 _= *π*_2 _= ... = *π*_*k *_against

*H*_1_: *π*_1 _≤ *π*_2 _≤ ... ≤ *π*_*k *_with at least one strict inequality.

For simplicity, assume increasing effects with increased exposure; analogously, a directional decision for a decrease is possible.

There are an extensive number of publications concerning order-restricted tests, including the analysis of 2 × *k *contingency tables (e.g. Agresti and Coull, [5]; Leuraud and Benichou, [15]). However, no uniformly powerful trend test exists for all possible alternative shapes. The possible shapes can be seen as different equality-inequality patterns of H_1_. This can be seen for an extreme convex shape {0, 0, 0, *π*}. Clearly, the "Helmert's contrast" is most powerful because of the optimal pooling of all the lower exposures and the comparison with the high exposure: *p*_4 _- (*p*_1 _+ *p*_2 _+ *p*_3_)/3. However, power for Helmert's contrast is greatly reduced for the extreme concave shape {0, *π*, *π*, *π*}. The shape of the exposure-response relationship is unknown a priori. Irrespective of numerous recent alternative proposals, the likelihood ratio test represents an appropriate solution for this situation. This test is numerically complicated, particularly concerning its distribution under the alternative, which is needed for power/sample size calculations (Robertson et al., [16]). The multiple contrast test according to Bretz and Hothorn [17] approximates its power and is simpler. There are *2*^*k*^*-1 *different shapes for *k *exposure groups, and for each shape a contrast with a minimum false negative rate (*β*) can be defined. The idea is to select the best contrast, which is sensitive for a certain shape. The best contrast is simply tested by a maximum test. Because the proportions *p*_*j *_are asymptotically normally distributed, their linear combination (denoted as contrast) $\sum_{j=1}^{k}c_{j}p_{j}$ is also normally distributed, and therefore, the single contrast test statistic $t_{SingleC}=\frac{\sum_{j=1}^{k}c_{j}p_{j}}{\sqrt{p(1-p)\sum_{j=1}^{k}c_{j}^{2}/n_{j.}}}$ is asymptotically normally distributed, where ∑_*j *_*c*_*j *_= 0 guarantees a level *α *test under the null hypothesis. Different variance estimators can be used, but to keep the problem simple, the commonly used pooled estimator *p *is used here. Asymptotic test versions are used throughout. The contrast coefficients, *c*_*j*_, are specific for each contrast test; for example the Helmert's contrast [*c*_*i *_= -1; *j *= 1,..., *k *- 1 and *c*_*k *_= *k*]. A multiple contrast test is the maximum of *s *pre-defined single contrast tests $t_{MultipleC}=\underset{i\in (1,...,s)}{\max}(t_{SingleC}(c_{i}))$, *i *= 1,..., *s *where **c**_**i **_= (*c*_*i*1_,..., *c*_*ik*_) is a *k *vector of contrasts. Under the null hypotheses, the joint distribution of the linear contrast tests *t*_*SingleC*_(**c**_*i*_) *i *= 1,..., *s *is an *s*-variate normal distribution with a zero vector of means and a non-product-moment correlation matrix. The correlation between two arbitrary contrasts, **a **= (*a*_1_,..., *a*_*k*_) and **b **= (*b*_1_,..., *b*_*k*_), is $\rho_{a,b}=\frac{\sum_{j=1}^{k}a_{j}b_{j}\frac{p_{j}(1-p_{j})}{n_{j.}}}{\sqrt{\left(\sum_{j=1}^{k}a_{j}^{2}\frac{p_{j}(1-p_{j})}{n_{j.}}\right)\left(\sum_{j=1}^{k}b_{j}^{2}\frac{p_{j}(1-p_{j})}{n_{j.}}\right)}}$.

This so-called isotonic contrast approach, based on s = 7 contrasts, for the balanced design with four exposure groups is demonstrated in Table 3.

**Table 3:** Contrast coefficients for the balanced design with four exposures groups

Type of contrasts	No. of contrasts	Alternative	Contrast *c*_*j*_
Isotonic	2^k^-1	*π*_1 _<*π*_2 _= *π*_3 _= *π*_4_	{-3 1 1 1}
		*π*_1 _= *π*_2 _<*π*_3 _= *π*_4_	{-1 -1 1 1}
		*π*_1 _= *π*_2 _= *π*_3 _<*π*_4_	{-1 -1 -1 3}
		*π*_1 _<*π*_2 _<*π*_3 _<*π*_4_	{-3 -1 1 3}
		*π*_1 _= *π*_2 _<*π*_3 _<*π*_4_	{-1 -1 0 2}
		*π*_1 _<*π*_2 _= *π*_3 _<*π*_4_	{-1 0 0 1}
		*π*_1 _<*π*_2 _<*π*_3 _= *π*_4_	{-2 0 1 1}
Change point	k-1	*π*_1 _<*π*_2 _= *π*_3 _= *π*_4_	{-3 1 1 1}
		*π*_1 _= *π*_2 _<*π*_3 _= *π*_4_	{-1 -1 1 1}
		*π*_1 _= *π*_2 _= *π*_3 _<*π*_4_	{-1 -1 -1 3}
Up/down	2	*π*_1 _<*π*_2 _= *π*_3 _= *π*_4_	{-3 1 1 1}
		*π*_1 _= *π*_2 _= *π*_3 _<*π*_4_	{-1 -1 -1 3}
Single (linear)	1	*π*_1 _<*π*_2 _<*π*_3 _<*π*_4_	{-3 -1 1 3}

However, the correct classification rates for the most likely elementary alternative (shape of the exposure-response) were found to be unsatisfactory for isotonic contrasts (Hothorn et al., [6]). Therefore, a special case of order-restricted inference is considered for step shapes only and denoted as a change point alternative (Hirotsu and Marumo, [18]). Two situations should be considered: i) threshold level studies assuming that an exposure-response reveals a single change point, which can be characterized by a lower part, an upper part, and an abrupt change between both; and ii) exposure-response studies with continuous exposure data where the change point alternative is a special and substantial component of the all-pattern alternative, which can simplify the evaluation. In some epidemiological problems this question arises. An example of a threshold level study is a diabetes study (Pastor-Barriuso et al., [19]) with the relationship between 2-hour plasma glucose and mortality, where the following questions were formulated: i) Does a certain glucose level exists that markedly increases the mortality risk? ii) Can this change point be estimated? Proposals in the literature are directed only at proof of the existence of such a change point. However, epidemiologists not only want to know that such a change exists, but also where this change is located. Here it is demonstrated that the estimation of the change point *q *is characterized by its correct classification rate by means of multiple contrast tests, that is, in a testing framework. The hypotheses system for a change from *q *to *q*+1 is:

*H*_0_: *π*_1 _= *π*_2 _= ... = *π*_*k*_

*H*_1_: *π*_1 _= ... = *π*_*q *_<*π*_*q*+1 _= ... *π*_*k *_   *q *∈ (**1**,..., *k *- **1**)

The above hypotheses system can be tested by multiple step contrasts. Exactly (*k-1*) step contrasts are appropriate for testing the above hypothesis:

$$\begin{array}{ccccc} (-k, & 1, & 1, & ... & 1)\\ (-(k-1), & -(k-1), & 2, & .... & 2)\\ ... & ... & ... & ... & ...\\ (-1, & -1, & -1, & ... & k) \end{array}$$

Exactly three possible change points, *q*, exist for the simple design with one unexposed and three exposure groups. Exactly one contrast is power-optimal for the balanced design of each change point:

$$\begin{array}{ccccc} q & c_{1} & c_{2} & c_{3} & c_{4}\\ 1 & (-3 & 1 & 1 & 1)\\ 2 & (-2 & -2 & 2 & 2)\\ 3 & (-1 & -1 & -1 & 3) \end{array}$$

"Power-optimal" simply means the maximum test statistics because the $t_{SingleC}^{i}$ is normally distributed, and therefore, standardized. The *t*_*MultipleC *_is *q*-variate normally distributed. The contrast coefficients, **c**, for *q *contrasts are defined for the general unbalanced design (Hirotsu et al., [20]):

$$c_{qj}=\{\begin{array}{lll} -n_{j.}/\sum_{l=1}^{j}n_{l.} & if & j=1,...,q\\ n_{j.}/\sum_{l=j}^{k}n_{l.} & if & j=q+1,...,k \end{array}$$

These step contrasts reveal a nice ability to transform the *k*-sample problem into an unbalanced two-sample problem, which can be used later for estimation of the unadjusted relative risk (or odds ratio) "above/below" the change point. Moreover, the step contrasts belong to a broader class of multiple contrasts. Isotonic contrasts approximate the power of the likelihood ratio test for the monotone ordered hypothesis. The bivariate up/down proposals (Neuhaeuser and Hothorn, [21]; Stewart and Ruberg, [22]) only use the two extreme contrasts (Table 3). Therefore, the change point alternative represents a compromise for testing trends. It is much less dependent on the power of the shape compared with the frequently used single linear contrast test, although only *k *instead of 2^*k *^- 1 isotonic contrasts were used. The multiple contrast test (above) is defined for differences of proportions, but can be re-formulated for the relative risk, commonly used in epidemiology (see Appendix A).

It seems that a multiple contrast test may be a different approach to the commonly used logistic model. However, a strong relationship between the multiple contrast test and the score test in a logistic model exists, which allows the correction for additional confounders (Hothorn et al., [6]).

### Identification of the exposure-response shape

The trend tests distinguish only globally between the null hypothesis and alternative hypotheses, based on the asymptotic distribution of the test statistics under the null hypothesis. That is, either a trend exists or it does not. However, the alternative hypothesis is not unique. For example, the following three hypotheses are possible for the change point alternative for a design with one unexposed and three exposure groups:

$$H_{1}^{1}:\pi_{1}<\pi_{2}=\pi_{3}=\pi_{4},\;H_{1}^{2}:\pi_{1}=\pi_{2}<\pi_{3}=\pi_{4},\;H_{1}^{3}:\pi_{1}=\pi_{2}=\pi_{3}<\pi_{4}$$

However, the global trend tests provide no answer as to which particular alternative exists. Two different approaches can be used to answer this question: i) the best contrast approach; and ii) a model selection approach based on the information criterion for order restriction. This paper explores the identification of one of the possible *k *- 1 elementary alternatives; that is, a classification into $H_{1}^{1},...,H_{1}^{k-1}$. Consequently, the correct classification rate, or the proportion of correctly identified elementary alternatives, is used as a performance measure later on.

The global test decision for the multiple contrast approach is based on the maximum of all included single contrasts $t_{MultipleC}=\underset{i\in (1,...,s)}{\max}(t_{SingleC}(c_{i}))$, *i *= 1,..., *s*, where each single contrast is power optimal for a particular type of alternative (Table 3). Therefore, this maximum contrast approach can be used as an estimator for the exposure-response shape, where the classification is performed after a significant trend test for control *α*. For example, two alternatives are possible for a design with three exposure groups: $H_{1}^{1}$*π*_1 _= *π*_2 _<*π*_3 _or $H_{1}^{2}$ : *π*_1 _<*π*_2 _= *π*_3_. Assume that the number of diseased cases, *n*_11_,..., *n*_*k*1_, is drawn from *k *binomial random variables with parameters *π*_*j *_and *n*_j. _A possible exposure-response is described by a contrast vector, **c **= (*c*_1_,..., *c*_*k*_). The problem is to estimate the underlying exposure-response relationship when *s *contrast vectors are given. A simple estimator is the function Ψ : (*n*_11_,..., *n*_*k*1_) → {1,..., *s*} which can be derived from the associated contrast test, i.e. $\psi_{1}=\psi (n_{11},...,n_{k1})=\underset{i\in (1,...,s)}{\arg\max}\left|t_{SingleC}(c_{i})\right|$. Then explore variability of the simple estimator, Ψ_1_. How likely is each of the *s *possible values under the observed data? This question can be addressed via the parametric bootstrap. Repeated realizations from *k *binomial distributions with sample sizes *n*_*j*_. and the estimated success parameter *p*_*j *_= *n*_*j*1_/*n*_*j*. _for j = 1,..., *k *are drawn.

• Draw B bootstrap samples $n_{11}^{*b},...,n_{k1}^{*b},\;n_{j1}^{*b}\sim B(n_{j.},p_{j}),\;b=1,...,B$

• Compute $\psi_{1}^{b}=\psi_{1}(n_{11}^{*b},...,n_{k1}^{*b})$

• Compute the relative frequency of each possible value from 1,..., s

This is a measure for the variance of the estimator. Under special circumstances, an improved estimator can be computed by a majority voting according to Breiman [23] over $\psi_{1}^{b}:\psi_{2}=\underset{i\in \{1,...,s\}}{\arg\max}\sum_{b}I(\psi_{1}^{b}=i)$, where *I *denotes the indicator function. This approach is designated the "parametric bootstrap best contrast" approach.

The model selection approach, based on the information criterion for order-restriction of normally distributed variables according to Anraku [14], can be modified for proportions and the change point alternative. The AIC criterion for the unrestricted maximum likelihood estimator $\hat{\theta }:AIC(\hat{\theta })=l(\hat{\theta })-p$: (with *l*($\hat{\theta }$) = log-likelihood, *p *= dimension of *θ*) was modified for order-restricted maximum likelihood estimators: $ORIC(\tilde{\theta })=l(\tilde{\theta })-penalty(k,n_{i})$. The penalty term is calculated for each model using the level of probabilities under an order-restriction. The explicit formulas for a design with three exposure groups, such as the null-model M_0 _and the two change point models M_1 _and M_2_, are given in Appendix B. The ORIC-approach represents a model estimation approach, where model *M*_0_{*H*_0_: *π*_1 _= *π*_2 _= *π*_3_}, model *M*_1 _{$H_{M_{1}}$ : *π*_1 _= *π*_2 _<*π*_3_}, or model *M*_2 _{$H_{M_{2}}$ : *π*_1 _= *π*_2 _<*π*_3_} will be estimated as a "best fitted" model.

### Simulation study

The simulation study is structured in two parts: i) empirical comparison between the best-contrast approach and the ORIC approach for a design with three groups; and ii) investigation of the best contrast approach for more general designs. Fifty thousand pseudo-random 2 × *k *tables (*k *ranging from *3 *to *7*) were generated and 10,000 bootstrap samples were drawn. Two criteria are used, the correct classification rate – the empirical decision rate for the correct model – and the power.

#### Part I

The correct classification rates for the ORIC approach, ORIC (M_0_, M_1_, M_2_), and the parametric bootstrap best contrast approach, Max(H^1^, H^2^), were compared for a design with three exposure groups (in Table 4) for the change point alternatives with different unexposed rates, *π*_1_. From the first row in Table 4, where no differences between the proportions were investigated, the main difference between both approaches becomes clear. The ORIC approach, as an estimation approach, did not control for *α*. Only in 76% of the cases, not 95%, was M_0 _selected under the null hypothesis. On the other hand, the best contrast test approach does control for *α*. Both approaches reveal high correct classification rates, greater than 90%, as long as the power is sufficient: either small unexposed rates, *π*_1_, or large non-centrality parameters Δ (Table I in Appendix C (available as additional file 1) and larger sample sizes in Table II in Appendix C). This behavior is similar to the power of trend tests of proportions (Bretz and Hothorn, [17]). Due to the fact that the correct classification rates of the best contrast approach are similar or superior to those of the ORIC approach with decreasing *π*_1_, increasing Δ, and n_j_, the best contrast approach is recommended because of its simplicity and generalizability for use within the generalized linear model.

**Table 4:** Correct classification rates for several spontaneous rates *π*_0_

***π***_***j***_	True Change *q*	ORIC(M_0_, M_1_, M_2_)			Max(H^1^, H^2^)	
		
		M_0_	M_1_	M_2_	H^1^	H^2^
*0.3/0.3/0.3*	*0*	*.758*	*.112*	*.129*	*.514*	*.486*
0.1/0.1/0.3	2	.001	**.979**	.021	**.987**	.004
0.1/0.3/0.3	1	.001	.020	**.980**	.030	**.961**
0.2/0.2/0.4	2	.002	**.958**	.041	**.936**	.023
0.2/0.4/0.4	1	.005	.029	**.967**	.040	**.926**
0.3/0.3/0.5	2	.006	**.940**	.054	**.906**	.034
0.3/0.5/0.5	1	.004	.053	**.943**	.044	**.882**
0.4/0.4/0.6	2	.009	**.940**	.052	**.887**	.036
0.4/0.6/0.6	1	.009	.053	**.940**	.039	**.885**

(n_j. _= 100, $H_{1}^{1}$ : *π*_0 _= *π*_1 _<*π*_2_, $H_{1}^{2}$ : *π*_0 _<*π*_1 _= *π*_2_) *(bold indicate correct classification)*

#### Part II

For one selected change point alternative {*π*_1_, *π*_1_, *π*_1_, *π*_1_, *π*_1 _+ Δ } the best contrast approach was investigated for the different dimensions *k*, different unexposed rates *π*_1_, and several non-centrality parameters Δ, shown in Table 5. With an increasing number of exposure groups, a slight decrease of the correct classification rate occurs where the power is slightly increasing. With a decreasing sample size, a slight decrease of the correct classification rate occurs where the power is substantially decreasing. The well-known decrease of sensitivity with an increasing unexposed rate from 2 × 2 table analysis holds true for power and, less markedly, for the correct classification rate. The effect size (non-centrality Δ) has much less impact on the correct classification rate compared with its well-known impact on power.

**Table 5:** Correct classification rates and power for several dimensions, sample sizes, unexposed rates, and non-centralities

Dimension	*k*	3	4	5	6	7
	Correct classif. rate	.992	.987	.977	.971	.971
	Power	*.828*	*.845*	*.861*	*.899*	*.889*
						
Sample size	n_j._	25	50	75	100	125
	Correct classif. rate	.809	.973	.978	.987	.989
	Power	*.393*	*.618*	*.742*	*.845*	*.903*
						
Unexpos. rate	*Π*_1_	.01	.06	.11	.16	.20
	Correct classif. rate	.987	.903	.817	.767	.766
	Power	*.845*	*.488*	*.373*	*.312*	*.266*
						
Non-centrality	Δ	0.03	0.05	0.07	0.09	0.11
	Correct classif. rate	*.953*	.973	.985	.994	.998
	Power	*.479*	.773	.904	.972	.991

Table 6 demonstrates the decreasing correct classification rate for change points *q *<<*k*. More important, from an epidemiological point of view, are the asymmetrical cumulative false classification rates. False classification is primarily from an overestimation instead of an underestimation of the true change point, that is, it is very unlikely to mistake a lower change point for the true one.

**Table 6:** Asymmetrical cumulative false classification rates

Alternative	True Change	H^1^	H^2^	H^3^	H^4^	H^5^	Cum. over.	Cum. under.
.01/.01/.01/.01/.01/.07	5	.000	.000	.001	.027	**.972**	-	0.028
.01/.01/.01/.01/.07/.07	4	.000	.002	.012	**.847**	.139	0.139	0.014
.01/.01/.01/.07/.07/.07	3	.000	.011	**.819**	.119	.051	0.17	0.011
.01/.01/.07/.07/.07/.07	2	.004	**.809**	.117	.038	.032	0.187	0.004
.01/.07/.07/.07/.07/.07	1	**.711**	.135	.052	.050	.053	0.29	-

(n_j _= 100; *bold indicate correct classification*)

### Extreme unbalanced exposure data

Particularly for environmental studies, much of the data is for unexposed and low-to-medium exposures; only rarely does data for high exposure exist. This is quite fortunate from an ethical point of view. However, this results in extremely unbalanced 2 × *k *tables and the statistical outcome depends on the rare, high-level exposure data. In a case-control study for respiratory cancer possibly caused by long-term exposure to coke oven emissions, the sample size was 10,198 in the unexposed group, but only 487 were in the highest exposure group (Costantino et al., [3]). A more extreme example was the study evaluating the connection between childhood cancer and magnetic fields from high voltage installations. The sample size was 2 in the highest exposure group, but 6,457 in the unexposed group (Table 1). The power decreases greatly for extremely unbalanced designs and accordingly the correct classification rate also decreases. If the total sample size is increased to achieve the same power, then the correct classification would be of the same magnitude as the balanced case, see Table 7. The identification of a trend in such a highly unbalanced design is complicated. A significant trend may depend on only these few cases, and the size and power of unbalanced designs differ greatly from those in balanced designs. In unbalanced designs with smaller change points, the correct classification rate increases if the resulting two-sample test is less unbalanced (as a result of the related step contrast). A change point at a high exposure that is based on rare data is very vague, however it becomes more stable when medium-to-high exposure from additional data are obtained.

**Table 7:** Correct classification rates for extreme unbalanced designs

Sample sizes	N	Alternative	Power	Correct classif. rate
200/200/200/200	800	.05/.05/.05/.10	.682	**.935**
540/200/40/20	800	.05/.05/.05/.10	*.251*	**.758**
200/200/200/200	800	.05/.05/.10/.10	.792	**.831**
540/200/40/20	800	.05/.05/.10/.10	*.425*	**.687**
200/200/200/200	800	.05/.10/.10/.10	.603	**.783**
540/200/40/20	800	.05/.10/.10/.10	.755	**.854**
400/400/400/400	1600	.05/.05/.05/.10	.915	**.971**
1340/200/40/20	1600	.05/.05/.05/.10	*.266*	**.749**
400/400/400/400	1600	.05/.05/.10/.10	.968	**.916**
1340/200/40/20	1600	.05/.05/.10/.10	*.438*	**.667**
400/400/400/400	1600	.05/.10/.10/.10	.903	**.904**
1340/200/40/20	1600	.05/.10/.10/.10	.832	**.883**

9740/200/40/20	10000	.05/.05/.05/.10	*.252*	**.702**

Unbalanced designs, where the smallest sample size occurs in the informative groups (large change point *s*), reveal a clearly reduced classification rate. However, that decrease, compared with the balanced design, is much weaker than the related power loss. A further reduction occurs for the "in-between" change points as long as the sample size of the pooled informative groups is still smaller than the lower exposure groups. A further substantial increase of the sample size for the unexposed group had almost no influence on the classification rate.

Since a sample size of *n*_*j *_= 1 is possible, in principle, for this approach, the impact of the continuous exposure categorization can be demonstrated quantitatively with respect to power and classification rate. When a single change point exists, the best approach is the categorization below or above this change point. The true alternative is never known a priori when dealing with real data. Therefore, appropriate categorization may be helpful and inappropriate categorization can greatly reduce the sensitivity.

The asymptotic power for the change point alternative is available (Bretz and Hothorn, [17]). Based on an R-code, the power can be calculated for an arbitrary sample size pattern, which shapes the exposure response and dimensions *k*. Power estimation for unbalanced designs can be found in [6] whereas a serious power loss can be observed when the sample size in the informative high exposure groups is very small compared with the sample size in the unexposed or low exposure groups.

### Evaluation of the example

The *p*-value for the global trend test (change point alternative) and the classification rate of the best contrast approach is determined using an implementation of the proposed procedures in R (R Development Core Team, [24]). The most likely change point, *q*, and simultaneous confidence intervals for the related change point contrasts can be calculated for the 2 × *k *contingency table data. A marginal confidence interval can be estimated for each elementary contrast because it represents a linear combination of the proportions *p*_*j*_. Simultaneous confidence intervals for the maximum of several contrasts can be estimated using a multivariate normal distribution. A detailed description for the estimation of simultaneous confidence intervals for several multiple contrast tests can be found in [25] where the particular problems for binomial data were described recently [26]. The software is available as the R library *bindosres *as additional file 2. This file can be installed in the private R program via "Install packages from local zip files",

The magnet field cancer data in Table 8 revealed a change point *q *= 8 with a classification rate of 0.74 (*p*-value for a global trend = 0.002). The cumulative false classification of 0.26 is nearly concentrated on *q *= 7. The maximum simultaneous lower confidence limit is for the sub-set [10 vs. {1, 2, 3, 4, 5, 6, 7, 8, 9}] and seems to be medically relevant with 0.563, but differs only a little from that of sub-set {10, 9} vs. {1, 2, 3, 4, 5, 6, 7, 8} that is related to the change point. The analysis of the continuous data using maximally selected rank statistics gave a cut-point of 0.45 *μ*Tesla^1^. However, above this cut-point only six cancer cases with an exposure of 0.51, 0.73, 1.0, 1.59, 1.66, and 1.72, and two cases without cancer with exposures 0.73 and 0.83 *μ *Tesla were available. A careful interpretation is recommended: i) the correct classification rate is not high, ii) a high change point was identified, iii) above the change point are only 4 of 6,491 cases, and iv) the spontaneous rate of 0.263 is rather high. More examples and their interpretation can be found in Hothorn et al., [6].

**Table 8:** Child cancer and magnetic fields

Exposure/*μ *Tesla	*j*	*p*_*j*_	Pattern	Lower confidence limit
0–0.05	1	0.263	{10,9,8,7,6,5,4,3,2} vs.1	-.716
0.051–0.101	2	0	{10,9,8,7,6,5,4,3} vs.{1,2}	-.410
0.101-0.15	3	0.4	{10,9,8,7,6,5,4} vs.{1,2,3}	-.327
0.151-.20	4	0.25	{10,9,8,7,6,5} vs.{1,2,3,4}	-.246
0.201-0.25	5	0.25	{10,9,8,7,6} vs.{1,2,3,4,5}	-.139
0.251-0.30	6	0	{10,9,8,7} vs.{1,2,3,4,5,6}	.108
0.301-0.35	7	0	{10,9,8} vs.{1,2,3,4,5,6,7}	.343
0.351-0.85	8	0.5	{10,9} vs.{1,2,3,4,5,6,7,8}	.534
		
0.851-1.6	9	1	10 vs.{1,2,3,4,5,6,7,8,9}	.563
>1.61	10	1		

## Conclusion

Trend tests for the analysis of 2 × *k *tables using epidemiological exposure data are described to identify the change point alternatives. Not only is the identification of a trend of interest important, but also the information regarding the particular types of alternatives. The best contrast approach for the multiple contrast test is useful for identifying the type of alternative or the change point, whereas a parametric bootstrap is suitable for an assessment of the variability. Both the bootstrapped best contrast and the ORIC approach are appropriate for different dimensions, non-centralities, sample sizes, and the unexposed group rates (due to the asymmetry in binomial testing). The consequences of unbalanced designs – of a large number in the unexposed or low exposure groups and a small number in the high exposure groups – can be calculated depending on the expected shape. Simultaneous confidence intervals for the change point alternative are also available.

Approaches that test a global trend in epidemiological exposure data and also provide information on the pattern of the exposure-response relationship are rare. The most competitive approach is the fractional polynomials model [7], which is a specific multivariable regression approach.

Most epidemiological studies are characterized not only by the primary exposure factor but also by several covariates, such as gender, age, occupational status, and competing risk characteristics. Therefore, the best contrast approach within the framework of the generalized linear model is recently available [27]. Using the related R library (multcomp), real data can be evaluated using the contrast option "Changepoint" [28].

The suitability of such a simple change point alternative in epidemiological exposure studies should be critically discussed and some real data examples tested. Clearly, such a change point test describes the exposure-response of the population only. Further investigations are required to demonstrate that this simple approach can be utilized to estimate the center of the individual-level change point distribution. Moreover, the above approach is not limited to change point alternatives: other trend alternatives, such as Williams-type trends [29], can be assumed as well.

## Competing interests

The authors declare that they have no competing interests.

## Authors' contributions

LAH adapted the multiple contrast tests on epidemiological case-control studies and performed part of the simulation study. MV selected, analyzed, and interpreted the epidemiological examples and designed the simulation study. TH developed the majority voting algorithm and wrote the R program.

## Appendix A

### Formulation contrast tests for the relative risk

The estimators for the relative risk (RR) of each exposure group versus unexposed (j = 1) are: $\begin{array}{cc} RR_{j1}=\frac{n_{j1}/n_{j.}}{n_{11}/n_{1.}} & j=2,...,k \end{array}$ The single contrast tests can be formulated for relative risks, for example for the reverse Helmert's contrast: $t_{revHelmert}=\frac{-kp_{1}+\sum_{j=2}^{k}p_{j}}{\sqrt{p(1-p)(\sum_{j=2}^{k}1/n_{j.}+k^{2}/n_{1})}}$

$$t_{revHelmert}^{RR}=\frac{-k\frac{n_{11}}{n_{1.}}+\sum_{j=2}^{k}\frac{n_{j1}}{n_{j.}}}{\sqrt{p(1-p)(\sum_{j=2}^{k}1/n_{j}.+k^{2}/n_{1})}}=\frac{-k+\sum_{j=2}^{k}\frac{n_{j1}}{n_{j.}}\frac{n_{1.}}{n_{11}}}{\frac{n_{1.}}{n_{11}}\sqrt{p(1-p)(\sum_{j=2}^{k}1/n_{j.}+k^{2}/n_{1})}}=\frac{-k+\sum_{j=2}^{k}RR_{j1}}{\frac{n_{1.}}{n_{11}}\sqrt{p(1-p)(\sum_{j=2}^{k}1/n_{j}+k^{2}/n_{1})}}$$

For general contrasts hold true $t_{SingleContrast}^{RR}=\frac{c_{1}+\sum_{j=2}^{k}c_{j}RR_{j1}}{\frac{n_{1.}}{n_{11}}\sqrt{p(1-p)(\sum_{j=1}^{k}c_{j}^{2}/n_{j.})}}$

## Appendix B

The ORIC approach for three binomials and the change point alternative. The three models are:

*M*_0_{*H*_0_: *π*_1 _= *π*_2 _= *π*_3_}, *M*_1 _{$H_{M_{1}}$ : *π*_1 _= *π*_2 _<*π*_3_}, *M*_2 _{$H_{M_{2}}$ : *π*_1 _<*π*_2 _= *π*_3_}. The likelihood is $L(\pi )=\frac{n_{1.}!}{n_{11}!(n_{1.}-n_{11})!}\pi_{1}^{n_{11}}{\left(1-\pi_{1}\right)}^{n_{1.}-n_{11}}\frac{n_{2.}!}{n_{21}!(n_{2.}-n_{21})!}\pi_{2}^{n_{21}}{\left(1-\pi_{2}\right)}^{n_{2.}-n_{21}}\frac{n_{3.}!}{n_{31}!(n_{3.}-n_{31})!}\pi_{3}^{n_{31}}{\left(1-\pi_{3}\right)}^{n_{3.}-n_{31}}$. With the expected values *π*_*j *_and their crude estimators: p1=n11n1., p2=n21n2., p3=n31n3.

The ${\tilde{p}}_{j}$ are the maximum likelihood estimates under the simple order restriction: ${\tilde{p}}_{j}=\underset{t:t\geq j}{\min}\underset{s:s\leq i}{\max}\frac{\sum_{j=s}^{t}w_{j}p_{j}}{\sum_{s=j}^{t}w_{j}}$. The likelihood for the null-model M_0 _is:

$L({\tilde{p}}_{H_{0}})=\prod_{j=1}^{3}\frac{n_{j.}!}{n_{j1}!(n_{j.}-n_{j1})!}{\tilde{p}}_{H_{0}}^{n_{11}+n_{21}+n_{31}}{\left(1-{\tilde{p}}_{H_{0}}\right)}^{n_{1.}+n_{2.}+n_{3.}-(n_{11}+n_{21}+n_{31})}$ where ${\tilde{p}}_{H_{0}}=\frac{n_{11}+n_{21}+n_{31}}{n_{1.}+n_{2.}+n_{3.}}=\frac{w_{1}p_{1}+w_{2}p_{2}+w_{3}p_{3}}{w_{1}+w_{2}+w_{3}}$ provided *w*_*j *_= *n*_*j*_

The likelihood for the model M_1 _is:

$$L({\tilde{p}}_{M_{1}})=\prod_{j=1}^{3}\frac{n_{j.}!}{n_{j1}!(n_{j.}-n_{j1})!}{\tilde{p}}_{\left(12\right)}^{n_{11}+n_{21}}{\left(1-{\tilde{p}}_{\left(12\right)}\right)}^{n_{1.}+n_{2.}-(n_{11}+n_{21})}{\tilde{p}}_{3}^{n_{31}}{\left(1-{\tilde{p}}_{3}\right)}^{n_{3.}-n_{31}}$$

where ${\tilde{p}}_{\left(12\right)}=\frac{n_{11}+n_{21}}{n_{1.}+n_{2.}}$, for $p_{\left(12\right)}<p_{3}\Rightarrow {\tilde{p}}_{12}=p_{\left(12\right)},{\tilde{p}}_{3}=p_{3}$

and ${\tilde{p}}_{\left(12\right)}\geq p_{3}\Rightarrow {\tilde{p}}_{12}={\tilde{p}}_{3}=\frac{w_{\left(12\right)}p_{\left(12\right)}+w_{3}p_{3}}{w_{\left(12\right)}+w_{3}}=\frac{n_{11}+n_{21}+n_{31}}{n_{1.}+n_{2.}+n_{3.}}$

The likelihood for the model M_2 _is:

$$L({\tilde{p}}_{M_{2}})=\prod_{j=1}^{3}\frac{n_{j.}!}{n_{j1}!(n_{j.}-n_{j1})!}{\tilde{p}}_{\left(23\right)}^{n_{21}+n_{31}}{\left(1-{\tilde{p}}_{\left(23\right)}\right)}^{n_{2.}+n_{3.}-(n_{21}+n_{31})}{\tilde{p}}_{1}^{n_{11}}{\left(1-{\tilde{p}}_{1}\right)}^{n_{1.}-n_{11}}$$

where ${\tilde{p}}_{\left(23\right)}=\frac{n_{21}+n_{31}}{n_{2.}+n_{3.}}$ for $p_{1}<{\tilde{p}}_{\left(23\right)}\Rightarrow {\tilde{p}}_{1}=p_{1},{\tilde{p}}_{\left(23\right)}={\tilde{p}}_{\left(23\right)}$

and $p_{1}\geq {\tilde{p}}_{\left(23\right)}\Rightarrow {\tilde{p}}_{1}={\tilde{p}}_{\left(23\right)}=\frac{w_{1}p_{1}+w_{\left(23\right)}{\tilde{p}}_{\left(23\right)}}{w_{1}+w_{\left(23\right)}}=\frac{n_{11}+n_{21}+n_{31}}{n_{1.}+n_{2.}+n_{3.}}$

The model-specific ORIC are: *ORIC*(*M*_*r*_) = log *L *(${\tilde{\pi }}_{M}$) - *penalty*(*M*_*r*_).

Where the penalty terms are $penalty(M_{r})=\sum iP\left\{i,j,w(M_{r})\right\}$

With

*w*(*M*_0_) = *n*_1. _+ *n*_2. _+ *n*_3._

*w*(*M*_1_) = *n*_1. _+ *n*_2._, *n*_3._

*w*(*M*_2_) = *n*_1._, *n*_2 _+ *n*_3._

Because *P*{1,1, *w*(*M*_0_)} = 1 *ORIC*(*M*_0_) = *L*($P\left\{1,1,w(M_{0})\right\}=1\;ORIC(M_{0})=L({\tilde{p}}_{H_{0}})-1$) - 1

Because $\begin{array}{ccc} P\left\{1,2,w(M_{1})\right\}=\frac{1}{2} & P\left\{2,2,w(M_{1})\right\}=\frac{1}{2} & ORIC(M_{1})=L({\tilde{p}}_{M_{1}})-\frac{3}{2} \end{array}$

Because $\begin{array}{ccc} P\left\{1,2,w(M_{2})\right\}=\frac{1}{2} & P\left\{2,2,w(M_{2})\right\}=\frac{1}{2} & ORIC(M_{2})=L({\tilde{p}}_{M_{2}})-\frac{3}{2} \end{array}$

## Supplementary Material

Additional file 1 **Additional simulation results.** Contains three tables with simulated correct classification results Click here for file

Additional file 2 **R package bindosres**. The R package bindosres which can be installed in R via "install packages from local zip files" Click here for file
