# Supplementary material for: Trend tests for the evaluation of exposure-response relationships in epidemiological exposure studies
Source: Epidemiol Perspect Innov. 2009 Mar 6;6:1. doi: 10.1186/1742-5573-6-1 (PMC2666731; doi:10.1186/1742-5573-6-1)
Supplement: Additional file 1 — Additional simulation results. Contains three tables with simulated correct classification results [file 1742-5573-6-1-S1.pdf]

Appendix C: Additional simulation results

TABLE I Correct classification rates for several non-centrality parameters

$\boldsymbol{\pi_j}$	ORIC(M_0,M_1,M_2)			Max(H^1,H^2)		
	M_0	M_1	M_2	power	H^1	H^2
0.3/0.3/0.35	.465	**.362**	.174	.177	**.739**	.261
0.3/0.3/0.4	.179	**.686**	.134	.472	**.861**	.139
0.3/0.3/0.45	.042	**.867**	.092	.764	**.918**	.082
0.3/0.3/0.5	.008	**.947**	.045	.940	**.906**	.034
0.3/0.3/0.55	.0005	**.978**	.022	.992	**.986**	.014
0.3/0.3/0.6	.000	**.991**	.009	.999	**.998**	.002

(bold indicate correct classification)

TABLE II Correct classification rates for several sample sizes n_j

$\boldsymbol{\pi_j}$	$n_{j.}$	True Change	ORIC(M_0,M_1,M_2)			Max(H^1,H^2)		
			M_0	M_1	M_2	Power	H^1	H^2
0.3/0.3/0.5	150	1	.0010	**.975**	.025	.991	**.983**	.017
0.3/0.5/0.5	150	2	.0005	.021	**.979**	.984	.020	**.980**
0.3/0.3/0.5	125	1	.001	**.972**	.028	.975	**.954**	.021
0.3/0.5/0.5	125	2	.002	.026	**.972**	.968	.028	**.939**
0.3/0.3/0.5	100	1	.006	**.940**	.054	.940	**.906**	.034
0.3/0.5/0.5	100	2	.004	.053	**.943**	.926	.044	**.882**
0.3/0.3/0.5	75	1	.024	**.908**	.069	.869	**.824**	.044
0.3/0.5/0.5	75	2	.025	.072	**.903**	.852	.048	**.804**
0.3/0.3/0.5	50	1	.067	**.828**	.105	.699	**.645**	.054
0.3/0.5/0.5	50	2	.064	.100	**.837**	.683	.061	**.622**
0.3/0.3/0.5	25	1	.196	**.644**	.160	*.450*	*.384*	*.065*
0.3/0.5/0.5	25	2	.199	.153	**.649**	*.427*	*.073*	*.354*

(bold indicate correct classification)

In practice the change point definition is relative to the pattern of proportions. In Table II the switch from $q = 3$ to $q = 2$ reveals a monotonic increase of the estimation of the alternative H^2. This increase is weaker for the switch from q=3 to q=1 according to the asymmetrical effect described in Table I.

TABLE III Correct classification rates for switching the change point

Alternative	Switch	Power	H^1	H^2	H^3
.01/.01/.01/.07	*q*=4→3	.862	.000	.021	**.979**
.01/.01/.02/.07		.809	.001	.114	.885
.01/.01/.03/.07		.809	.001	.268	.731
.01/.01/.04/.07		.820	.005	.454	.541
.01/.01/.05/.07		.851	.004	.629	.367
.01/.01/.06/.07		.885	.002	.770	.229
.01/.01/.07/.07		.907	.004	**.843**	.153
.01/.01/.01/.07	q=4→2	.862	.000	.021	**.979**
.01/.02/.02/.07		.727	.009	.074	.917
.01/.03/.03/.07		.639	.084	.156	.761
.01/.04/.04/.07		.603	.222	.188	.590
.01/.05/.05/.07		.599	.422	.241	.337
.01/.06/.06/.07		.662	.610	.196	.193
.01/.07/.07/.07		.728	**.764**	.147	.089

(n_j=100; bold indicate correct classification)
